# Autonomic nervous system markers of music-elicited analgesia in people with fibromyalgia: A double-blind randomized pilot study

**DOI:** 10.3389/fpain.2022.953118

**Published:** 2022-09-15

**Authors:** Rebecca J. Lepping, Miranda L. McMillan, Andrea L. Chadwick, Zaid M. Mansour, Laura E. Martin, Kathleen M. Gustafson

**Affiliations:** ^1^Hoglund Biomedical Imaging Center, University of Kansas Medical Center, Kansas City, KS, United States; ^2^Department of Anesthesiology, Pain, and Perioperative Medicine, University of Kansas Medical Center, Kansas City, KS, United States; ^3^Department of Physical and Occupational Therapy, The Hashemite University, Zarqa, Jordan; ^4^Department of Population Health, University of Kansas Medical Center, Kansas City, KS, United States; ^5^Department for McMillan and Chadwick, Cofrin Logan Center for Addiction Research and Treatment, University of Kansas, Lawrence, KS, United States; ^6^Department of Neurology, University of Kansas Medical Center, Kansas City, KS, United States

**Keywords:** pain, auditory distraction, quantitative sensory testing, music, nature sounds

## Abstract

**Purpose:**

To investigate the feasibility of using music listening by adults with fibromyalgia (FM) as a potential tool for reducing pain sensitivity.

**Patients and methods:**

We report results from a double-blind two-arm parallel randomized pilot study (NCT04059042) in nine participants with FM. Pain tolerance and threshold were measured objectively using quantitative sensory tests; autonomic nervous system (ANS) reactivity was measured with an electrocardiogram. Participants were randomized to listen to instrumental Western Classical music or a nature sound control to test whether music listening elicits greater analgesic effects over simple auditory distraction. Participants also completed separate control testing with no sound that was counterbalanced between participants.

**Results:**

Participants were randomized 1:1 to music or nature sounds (four Music and five Nature). Although the groups were not different on FM scores, the Music group had marginally worse temporal pain summation (*p* = 0.06), and the Nature group had higher anxiety scores (*p* < 0.05). Outcome measures showed a significant difference between groups in the magnitude of change in temporal summation between sessions (*p* < 0.05), revealing that the Nature group had greater pain reduction during audio compared to silence mode, while the Music group had no difference between the sessions. No significant effects were observed for either mechanical pain tolerance or ANS testing. Within the Music group, there was a trend of vagal response increase from baseline to music listening, but it did not reach statistical significance; this pattern was not observed in the Nature group.

**Conclusion:**

Auditory listening significantly altered pain responses. There may be a greater vagal response to music vs. nature sounds; however, results could be due to group differences in pain and anxiety. This line of study will help in determining whether music could be prophylactic for people with FM when acute pain is expected.

## Introduction

The term “centralized pain” describes any central nervous system (CNS) dysfunction or pathology that may contribute to the development or maintenance of chronic pain (1–3). There is a growing appreciation of the role of CNS augmentation in pain processing in many chronic pain conditions (2, 4). A hallmark of the centralized pain phenotype is the presence of hyperalgesia and reduced or absence of endogenous analgesia (5–7). Data from quantitative sensory testing (QST) studies suggest a wide, bell-shaped distribution in pain sensitivity across the general population. Most individuals with centralized pain fall on the right side of this curve and have QST findings consistent with hypersensitivity (hyperalgesia and allodynia) (1, 8–13). QST evidence of widespread hypersensitivity is consistently observed in many chronic pain conditions, including FM, irritable bowel syndrome, tension headache, low back pain, temporomandibular joint disorder, interstitial cystitis, and vulvodynia (14–23). Widespread hypersensitivity is often measured through QST sensitivity testing of pain to pressure on the thumbnail bed. As evidence suggests, temporal summation, which is the phenomenon of amplifying pain perception after being subjected to repeated or continuous noxious stimulation, despite having the same intensity of the stimulus (24), is an essential role player in FM (25, 26). Therefore, in this study, we used QST to objectively measure pain sensitivity and temporal summation while listening to music compared to listening to nature sounds in patients with FM.

Music has been previously shown to influence parameters of the autonomic nervous system associated with anxiety (27), such as slowing heart rate (28) and respiration (29). Music listening can also reduce acute pain during surgery (30), post-operative recovery (31), orthodontic procedures (32), orthopedic rehabilitation (33), and during thermic pain induction in healthy participants (34, 35). The *subjective* analgesic, anxiolytic, and antidepressant effects of music for people with chronic pain were recently confirmed in a meta-analysis (36). However, the impact of music listening on objective measures of pain sensitivity in patients with chronic pain has not yet been described. The goal of this pilot study was to understand the possible analgesic effects of music listening on objective measures of pain sensitivity in patients with fibromyalgia (FM).

The analgesic effect of music is thought to occur through several mechanisms: Contextual, Cognitive, Emotional, and Physiological (37, 38). First, music provides a predictable *context* that can increase the listeners' sense of control. This is further enhanced if the music is familiar, as this can bring in other effects that are not related to aspects of music specifically, such as setting up expectations and heightening nostalgia. Studies have shown the greatest analgesic effects when music is selected by participants. Second, similar to other types of stimulation, such as reading or listening to nature sounds (39), music can serve as a *cognitive* distraction and take attention away from the painful stimulus. Third, music is a powerful inducer of *emotion* (40, 41). Music that is positive, liked by the listener, and low on arousal has the strongest analgesic effect (34). Finally, music listening interventions and music therapy have also been shown to reduce anxiety and depression (42, 43). The anxiolytic effect may be due to the *physiological* effect of music on the parasympathetic nervous system, increasing the vagal response and reducing heart rate and respiration rate (27). Music also has effects on the brain directly, causing the release of endogenous opioids and dopamine and activating the areas of the descending pain modulatory system (44, 45). The specific musical characteristics that yield the greatest analgesic effects are difficult to pinpoint, as there is no standard for reporting. Meta-analyses have revealed that music with 60–80 beats per minute, in a major key, and without lyrics or percussion has the largest effects (46).

Previous studies in patients with FM have shown that patients have reduced self-reported pain and increased mobility after even a short, 10-min music listening intervention. After listening to the music of their choice, participants were faster in a standard mobility assessment, that is, the timed-up-and-go task (47). A second study using resting-state functional magnetic resonance imaging confirmed the impact of 5-min music listening intervention on the centralized descending pain modulatory system (DPMS), identified as changes in functional connectivity between regions of the DPMS that positively correlated with changes in pain scores (48). To our knowledge, this is the first study to investigate whether objectively measured pain sensitivity is reduced by music listening in patients with FM.

The goal of the current study was to identify whether music listening has a promising analgesic effect during pain threshold and tolerance testing for patients with FM that supersedes any effect of auditory distraction. We used standardized music, rather than music selected by the participants, so that we could determine whether the specific music characteristics described above (i.e., slow tempo, consonant harmonies, no lyrics, or percussion) would be sufficient to elicit an analgesic effect. While a personalized choice might elicit a greater effect, it would not be possible to determine whether the effect was due to the music characteristics or from the person's previous associations and memories with that music. We hypothesized that because the nature listening condition provides a distraction from pain sensations, and may also provide some of the same Contextual, Cognitive, Emotional, and Physiological impacts as music, both listening conditions (Music and Nature) would reduce pain sensitivity compared to testing during silence. However, as noted previously, the emotional and physiological impacts are anticipated to be stronger in music due, in part, to temporal structure and expectancy building. Therefore, we hypothesized that music listening would reduce pain sensitivity compared to nature sounds. We further hypothesized that music would increase vagal input to the autonomic nervous system, decreasing heart rate and increasing heart rate variability compared to both silence and nature sounds, and that analgesic responsiveness would be moderated by symptoms of FM, anxiety, and depression.

## Materials and methods

### Participants

Participants with a diagnosis of FM were recruited from pain clinics located at a large Midwestern US university medical center and by word of mouth. Eligible participants were 18 years or older, able to read and speak English, willing to refrain from alcohol, nicotine, and physical activity or exercise on the day of testing, and on a stable dose of adjunctive pain medications, including tricyclic antidepressants, serotonin-norepinephrine reuptake inhibitors, and gabapentinoids. Participants were excluded if they were not able to provide written consent, were pregnant, had peripheral neuropathy in the upper extremities, and had a severe physical impairment or co-morbid medical conditions, such as blindness, deafness, paraplegia, cancer, autoimmune disorder, liver failure or cirrhosis, hepatitis, cardiovascular disease, illicit drug or opioid abuse, or average daily opioid dosing of >15 mg oral morphine equivalents (e.g., > two 5 mg oxycodone tablets/day or >three 5 mg hydrocodone tablets/day). Conversions were made based on well-accepted conversion tools (49, 50).

**Figure 1:**
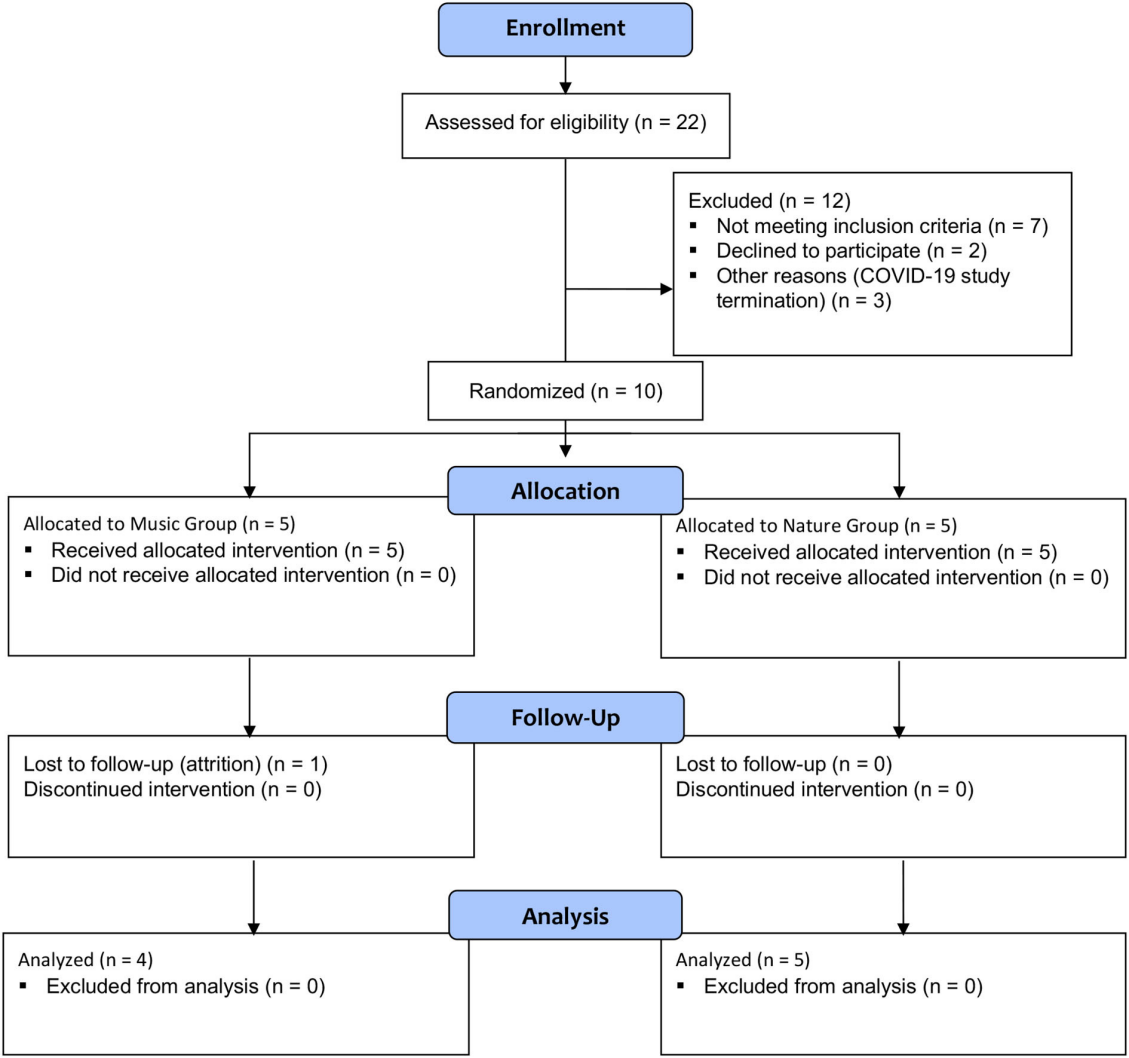
CONSORT 2010 flow diagram.

Ten White female participants with FM were enrolled in the study (Table 1). Centralized pain and nearly any chronic pain condition are 1.5–2 times more common in women than in men (51). One person in the Music group did not return for the second visit and was lost to follow-up. That person only received the silence session and was not included in the analysis (Figure 1).

**Table 1:** Participant demographic variables by audio group assignment (Music, Nature).

	**Music group (*n* = 4)**	**Nature group (*n* = 5)**	***U*/*X*^2^**	***p***
Age (years) [*M* (*SD*)]	49.18 (13.86)	40.28 (9.93)	6.00	0.41
Dominant hand, right [*n* (%)]	4 (100%)	5 (100%)	–	–
Gender, female	4 (100%)	5 (100%)	–	–
Sex assigned at birth, female	4 (100%)	4 (80%)	0.90	0.34
Race, white	4 (100%)	5 (100%)	–	–
Ethnicity: not Hispanic or Latinx	2 (50%)	5 (100%)	3.21	0.20
Hispanic or Latinx	1 (25%)	0 (0%)		
Other/unknown/no response	1 (25%)	0 (0%)		
Relationship status: married	2 (50%)	4 (80%)	3.60	0.31
Never married	1 (25%)	0 (0%)		
Divorced or separated	1 (25%)	1 (20%)		
Education: high school/GED	0 (0%)	0 (0%)	3.94	0.27
Some college	1 (25%)	1 (20%)		
Technical/associate's degree	0 (0%)	1 (20%)		
Bachelor's degree	0 (0%)	2 (40%)		
Advanced/professional degree	3 (75%)	1 (20%)		

Continuous measures were assessed with independent samples Mann–Whitney U-tests; categorical variables were assessed with Chi-square tests.

CI, confidence interval; M, mean; SD, standard deviation; GED, general education development.

The intended sample size was 40 participants with FM based on power analysis; however, due to the COVID-19 pandemic, recruitment was stopped and only 10 participants took part in this study.

### Measures

#### Participant self-report measures related to pain and music

##### Demographics

Participants completed a demographics questionnaire that included questions on participant sex, gender, age, race, ethnicity, marital status, education level, body mass index, and current medications.

##### Fibromyalgia-ness

Fibromyalgia-ness (FMness) is a measure of pain and co-morbid symptom extensiveness and severity, calculated by combining the scores of the Widespread Pain Index with the Symptom Severity Scale from the 2011 FM Survey (52) to derive a continuous metric purportedly indicative of the degree of CNS pain amplification present in a given individual (53).

##### Clinical pain severity

Pain severity and functional interference due to pain were assessed using the Brief Pain Inventory (BPI). The BPI is validated for chronic, non-malignant forms of pain, and asks patients to rate their current pain intensity, as well as their worst, least, and average pain in the 7 days (0–10 NRS), and has been recommended by IMMPACT as a measure of choice for the assessment of pain in clinical research (54–56).

##### Fibromyalgia functional status

Current health and functional status in FM patients were measured using the Revised Fibromyalgia Impact Questionnaire (FIQR) (57). The FIQR measures physical functioning, work status, and overall wellbeing.

##### Depression and anxiety

Mood symptoms were assessed with the static short forms for depression and anxiety, developed by the NIH roadmap initiative PROMIS (58). The PROMIS measures have a standardized mean of 50, a standard deviation of 10, and a range of 1–100.

##### Music experience

Participants rated their music listening habits (i.e., frequency, styles, reasons for listening, etc.) using the Brief Music Experience Questionnaire (MEQ) (59). The Brief MEQ is a 53-item self-report measure of music centrality in the respondent's life, their musical aptitude, and experience with and reaction to music. Questions are rated using a 5-point Likert scale (1: very untrue and 5: very true), from which six summary scores are derived for Commitment to Music, Innovative Musical Aptitude, Social Uplift, Affective Reactions to Music, Positive Psychotropic Effects from Music, and Reactive Musical Behavior.

#### Autonomic nervous system activity (ECG)

The study participants' ECG data were recorded using three standard snap-on ECG electrodes with Biopac MP150 and Acqknowledge 4.3 software (Goleta, CA). ECG electrodes were placed under the collar bone and below the rib cage on the opposite side, with a ground electrode placed on the abdomen near the navel. The time of each condition (baseline, listening only, and pain while listening) was recorded by the investigator with a mark in the Acqknowledge recording. The ECG data were uploaded to Kubios software (Kuopio, Finland) for analysis. Summary metrics of heart rate and variability during each condition were corrected for within-session baseline levels and compared between conditions (listening only vs. pain while listening) and between auditory groups (music vs. nature sounds).

#### Quantitative sensory testing (QST)

Pain testing was performed using the Multimodal Automated Sensory Testing (MAST) system, a computerized QST device developed at the University of Michigan and currently being employed in several clinical trials, including the NIH MAPP Network. Two measures of QST were used in this study: mechanical pain sensitivity (MPS) and temporal summation (TS). MPS was assessed by applying discrete pressure stimuli to the thumbnail bed. The MAST system delivered an ascending series of 5-s duration stimuli at 25-s intervals, beginning at 0.50 kg/cm^2^ and increasing in 0.50 kg/cm^2^ intervals up to tolerance or a maximum of 10 kg/cm^2^. Participants rated pain intensity after each stimulus on a 0 (no pain) – 100 (extreme pain) numerical rating scale (NRS). Pain threshold, the point at which participants rated >0 pain, and tolerance, the point at which participants rated >80 pain, were determined from this procedure. To measure TS, a 256 mN pinprick stimulus (MRC Systems, Heidelberg, Germany) was applied once to the forearm or hand, followed by a train of 10 identical stimuli at a rate of 1 Hz. Following the single stimulus and the train of 10 stimuli, patients reported the pain intensity of the pinprick sensation using the 0–100 NRS. This procedure was repeated three times, and the mean pain rating of the three stimulus trains was divided by the mean pain rating of the single stimuli to calculate a wind-up ratio (WUR); a WUR >1 indicates temporal summation (60).

### Stimuli and procedures

#### Music and sound delivery

Auditory stimuli were presented using a digital music player and noise-canceling headphones. Four audio tracks were identified by number only, and the researcher was blinded to the contents of each tract. One track was music, one was nature sounds, and two were silence modes. The randomization procedure indicated to the researcher which track (1–4) should be used for the testing session. Each track began with instructions to the participant, indicating what they would hear during testing, and that they should continue to wear the headphones even if the track is silent so that the researcher would not know what they were hearing.

#### Music characteristics

The musical selections consisted of professional recordings of instrumental Western classical music selected by the researcher (Supplementary Table 1). All participants heard the same pieces in the same order. Instrumentation ranged from piano solo to full orchestra, but all were without lyrics or heavy percussion. Pitch ranged across pieces but was standard across participants and not controlled by either the participant or the researcher. The tempo for all pieces was slow (~60 beats per minute). The pieces were in either major keys or minor keys, but all consisted primarily of consonant harmonies and sustained melodic phrases. Participants were allowed to control the volume to their individual comfort level.

#### Active control

Professional recordings of nature sounds (including forest, river, and wind sounds and birdsong) selected by the researcher without added music were used as the active control condition (Supplementary Table 1). All participants heard the same recording. This active control condition allowed for non-musical analgesic effects, such as distraction, to be controlled in the experimental design. Participants were allowed to control the volume to their individual comfort level.

#### Trial design

This was a single-center, two-arm parallel double-blind randomized controlled pilot study conducted in the United States (ClinicalTrials.gov, NCT04059042). Participants with FM underwent two testing sessions conducted 1 week apart: testing as usual with no sound (Silence), and testing while listening to instrumental Western classical music or nature sound control (Audio). Participants were randomized 1:1 to the two arms (Music or Nature sounds), counterbalanced for session order. Study data were collected and managed, and randomization was implemented using REDCap electronic data capture tools (61, 62).

#### Procedures

The study was conducted at a research laboratory within the medical center campus. Data were collected with participants seated in a small, quiet room across a small table from the researcher. The study team was blinded throughout data collection and analysis.

Participants in both arms had QST and electrocardiogram (ECG) testing on two separate days, conducted 1 week apart: baseline (Testing as Usual, Silence) and auditory listening (Music or Nature sounds) counterbalanced across participants. After obtaining informed consent, participants were fitted with ECG electrodes and were given instructions about the procedures. Participants were asked to wear noise-canceling headphones during all testing procedures, regardless of what they were hearing (music, nature sounds, or silence). The researcher wore ear plugs to remain blinded to what the participant was hearing and communicated with the participant through written instructions and gestures for the remainder of the test. Informed consent, instructions, and electrode placement took ~30 min. After the electrodes and headphones were in place, the researcher left the room, and baseline ECG was recorded for 5 min while participants sat quietly. The researcher returned to the room, started the specified audio track, and then left the room for 10 min while participants sat quietly listening to the track. The researcher then returned to the room for QST testing while the participant continued to listen to the audio track. QST procedures lasted for 15 min. Written instruction reminders were provided to participants before each task. At the end of the first day of testing, participants completed surveys electronically for 30 min on a laptop through REDCap (61, 62). All sessions were conducted in the same way and lasted approximately the same amount of time. The total testing time was 1.5 h on the first day of testing and 1 h on the second day of testing. After completing all procedures on the second day of testing, participants were given $100 for their time.

#### Randomization sequence generation

Participants were randomized 1:1 to Music or Active Control (Nature sounds), counterbalanced for session order with Silence. Randomization was implemented with the REDCap Randomization tool (61, 62) using an order defined by a computer-generated online random number generator for the four possible session orders (Music/Silence, Silence/Music, Nature/Silence, and Silence/Nature), coded by track number only, and was stratified by gender.

#### Randomization allocation/concealment method and implementation

Audio tracks for Music, Nature sounds, and two tracks for Silence were labeled with dummy codes (1–4) to blind the researcher collecting the data. The original audio tracks were given to a person outside the study team who renamed the files and placed the code into a sealed opaque envelope. The researcher selected the track by a number assigned during the randomization procedure. Randomization was concealed from the researchers until the final group analysis.

### Statistical analysis

Data were assessed for normality with tests for skewness and kurtosis (63). These tests revealed that several outcome variables had a non-normal distribution with skewness > |1| and kurtosis > |3| (Supplementary Table 2, Supplementary Figure 1). Therefore, non-parametric tests were conducted to compare groups and sessions (64). Demographic characteristics and questionnaire measures were compared between the two Audio Groups using independent samples Mann–Whitney *U*-tests for continuous variables and Chi-square (*X*^2^) test for categorical variables.

Pain outcome measures of temporal summation and mechanical pain tolerance were assessed using independent samples Mann–Whitney *U*-tests for between-group comparisons (Audio Group: Music, Nature) and related-samples Wilcoxon signed-rank test for within-subject comparisons (Sessions: Silence, Audio). To compare group differences in change in outcome measures across sessions, a magnitude of change score was calculated for each participant to reflect the degree of analgesia experienced during the Audio condition. For pain measures of temporal summation, for which higher values indicate worse pain, the score was calculated as Silence minus Audio; for mechanical pain tolerance, for which lower values indicate worse pain, the magnitude of change score was calculated as Audio minus Silence. Independent samples Mann–Whitney *U*-tests were then conducted for the magnitude of change scores for pain measures of temporal summation and mechanical pain tolerance.

The ANS measures of heart rate and heart rate variability (root mean square of successive differences, HRV) during listening and pain, corrected for baseline values, were assessed with independent samples Mann–Whitney *U*-tests for between-group comparisons (Audio Group: Music, Nature) and related-samples Wilcoxon signed-rank test for within-subject comparisons (Sessions: Silence, Audio). The magnitude of change score was calculated as Pain minus Listen to determine the within-session change during painful stimulation, and a second score was calculated as Pain minus Listen and Audio minus Silence to determine the change in analgesic effect across the sessions for each participant. The Pain minus Listen within-session magnitude of change was compared for within-subject comparisons between sessions (Silence, Audio) using related-samples Wilcoxon signed-rank test. To determine whether the Audio Groups (Music, Nature) differed in analgesic effect during pain, the magnitude of change score for Pain minus Listen and Audio minus Silence was compared using independent samples Mann–Whitney *U*-test. Statistical significance was set at *p* < 0.05 for each test.

## Results

### Demographic and questionnaire measures

Group differences in demographic measures are presented in Table 1. The groups did not differ in age, gender, ethnicity, relationship status, or education level. Questionnaire measures are presented in Table 2. Participants in both groups were experiencing moderate FM, depression, and anxiety symptoms. They also reported low to moderate commitment to music and innovative musical aptitude, but reported moderate to high affective reactions to music, positive psychotropic effects from music, and reactive musical behavior. The groups did not differ in FM symptom severity or musical experience; however, they were significantly different in symptoms of anxiety, with participants in the Nature group experiencing higher anxiety than participants in the Music group.

**Table 2:** Participant reported clinical and musical experience variables by audio group assignment (Music, Nature).

	**Music group (*n* = 4)**	**Nature group (*n* = 5)**	***U***	***p***
WOLFE FMness [*M* (*SD*)]	17.00 (4.76)	13.00 (3.74)	5.00	0.29
BPI worst 2 average	4.25 (2.63)	4.20 (1.64)	11.00	1.00
FIQR score	45.50 (22.19)	40.23 (15.78)	7.00	0.56
PROMIS: depression	45.98 (5.30)	50.40 (5.79)	12.00	0.73
PROMIS: anxiety	52.65 (4.71)	59.92 (3.33)	20.00	0.02^*^
MEQ: commitment to music	2.04 (0.92)	1.60 (0.71)	7.00	0.56
MEQ: innovative musical aptitude	2.11 (0.63)	2.17 (1.08)	9.50	0.91
MEQ: social uplift	2.62 (0.48)	2.70 (1.22)	9.00	0.91
MEQ: affective reactions to music	4.03 (0.84)	4.40 (0.33)	12.50	0.56
MEQ: positive psychotropic effects from music	3.53 (1.00)	3.45 (0.58)	11.00	1.00
MEQ: reactive musical behavior	3.58 (0.57)	4.00 (0.66)	14.00	0.41

Continuous measures were assessed with independent samples Mann–Whitney U-tests.

* Indicates significant group differences at p < 0.05.

CI, confidence interval; M, mean; SD, standard deviation; FM, fibromyalgia; BPI, Brief Pain Inventory; FIQR, Fibromyalgia Impact Questionnaire–Revised; PROMIS, Patient-Reported Outcomes Measurement Information System; MEQ, Music Experience Questionnaire.

### Pain measures

In the non-parametric tests for temporal summation, the difference between a single stimulus and a series of stimuli, the independent samples Mann–Whitney *U*-test identified a significant group difference in the magnitude of temporal summation between session changes (*p* = 0.03), with the Nature group showing lower temporal summation while listening to the audio compared to silence, while the Music group was not different between the sessions. The related-samples Wilcoxon signed-rank test for session revealed a non-significant trend (*p* = 0.051), with lower temporal summation during audio compared to silence. The independent samples Mann–Whitney *U*-test showed that temporal summation was marginally higher but not significantly different in the Music group compared to the Nature group (*p* = 0.06), indicating that participants in the Music group may have had higher temporal summation. Mechanical pain tolerance, the amount of pressure on the thumb that was rated at >80, was not significantly different between groups or between sessions (Table 3).

**Table 3:** Pain variables by session (Audio, Silence) and audio group assignment (Music, Nature).

	**Music group (*n* = 4) [*M* (*SD*)]**	**Nature group (*n* = 5) [*M* (*SD*)]**	**Statistical test**	***Z (p)***
Temporal summation: audio	20.25 (14.29)	4.13 (5.60)	–	–
Temporal summation: silence	20.17 (13.14)	9.40 (7.44)	–	–
Session difference: audio vs. silence	–	–	Related-samples Wilcoxon signed-rank	39.00 (0.051^†^)
Group difference: silence	–	–	Independent samples Mann–Whitney *U*	2.00 (0.06^†^)
Group difference: audio	–	–	Independent samples Mann–Whitney *U*	2.00 (0.06^†^)
Group difference in between session change	–	–	Independent samples Mann–Whitney *U*	19.00 (0.03^*^)
Mechanical pain tolerance: audio	4.12 (1.02)	5.17 (0.85)	–	–
Mechanical pain tolerance: silence	4.18 (1.00)	5.05 (0.94)	–	–
Session difference: audio vs. silence	–	–	Related-samples Wilcoxon signed-rank	18.00 (0.59)
Group difference: silence	–	–	Independent samples Mann–Whitney *U*	14.00 (0.41)
Group difference: audio	–	–	Independent samples Mann–Whitney *U*	16.00 (0.19)
Group difference in between session change	–	–	Independent samples Mann–Whitney *U*	14.00 (0.41)

Temporal summation is the difference in pain rating out of 100 between a single stimulus and the series of 10 in the non-dominant forearm. Mechanical pain tolerance is the pressure intensity (kg/cm^2^) at which participants rated pain in their non-dominant thumb at 70 out of 100. Between-session pain measures were assessed with related-samples Wilcoxon signed-rank tests. Between-group comparisons were assessed with independent samples Mann–Whitney U-tests. Between-session change scores were calculated per participant as Audio minus Silence and compared between groups with independent samples Mann–Whitney U-tests.

* Indicates significant effects at p < 0.05.

† Indicates non-significant effects at p < 0.10.

M, mean; SD, standard deviation.

### ANS measures

**Figure 2:**
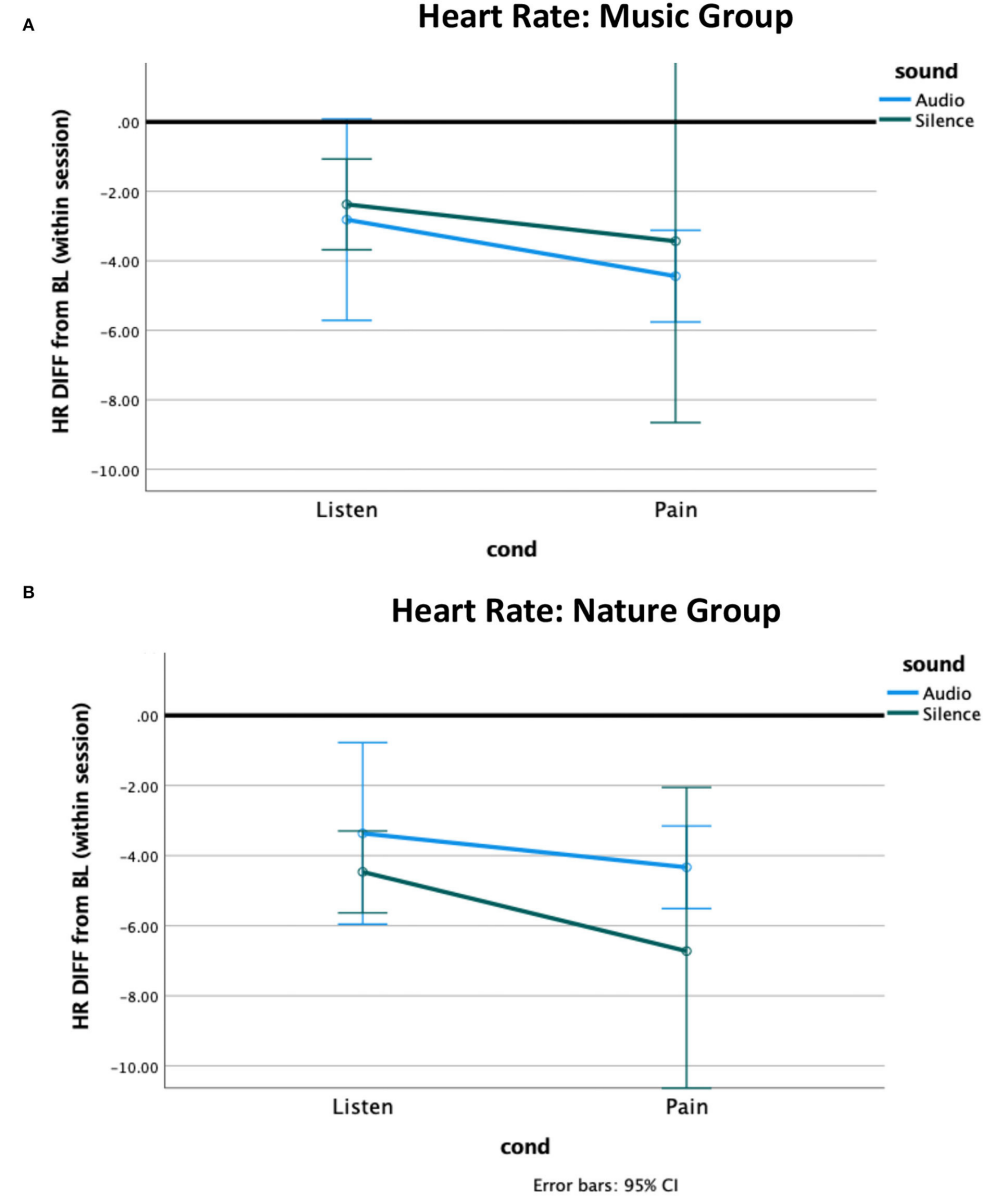
Heart rate difference from within-session baseline. The heart rate of both groups decreased from baseline to the listening condition and further decreased during pain. **(A)** The Music group had a greater pain-related decrease to music compared to silence, and **(B)** the Nature group had a greater pain-related decrease to silence compared to nature sounds.

**Figure 3:**
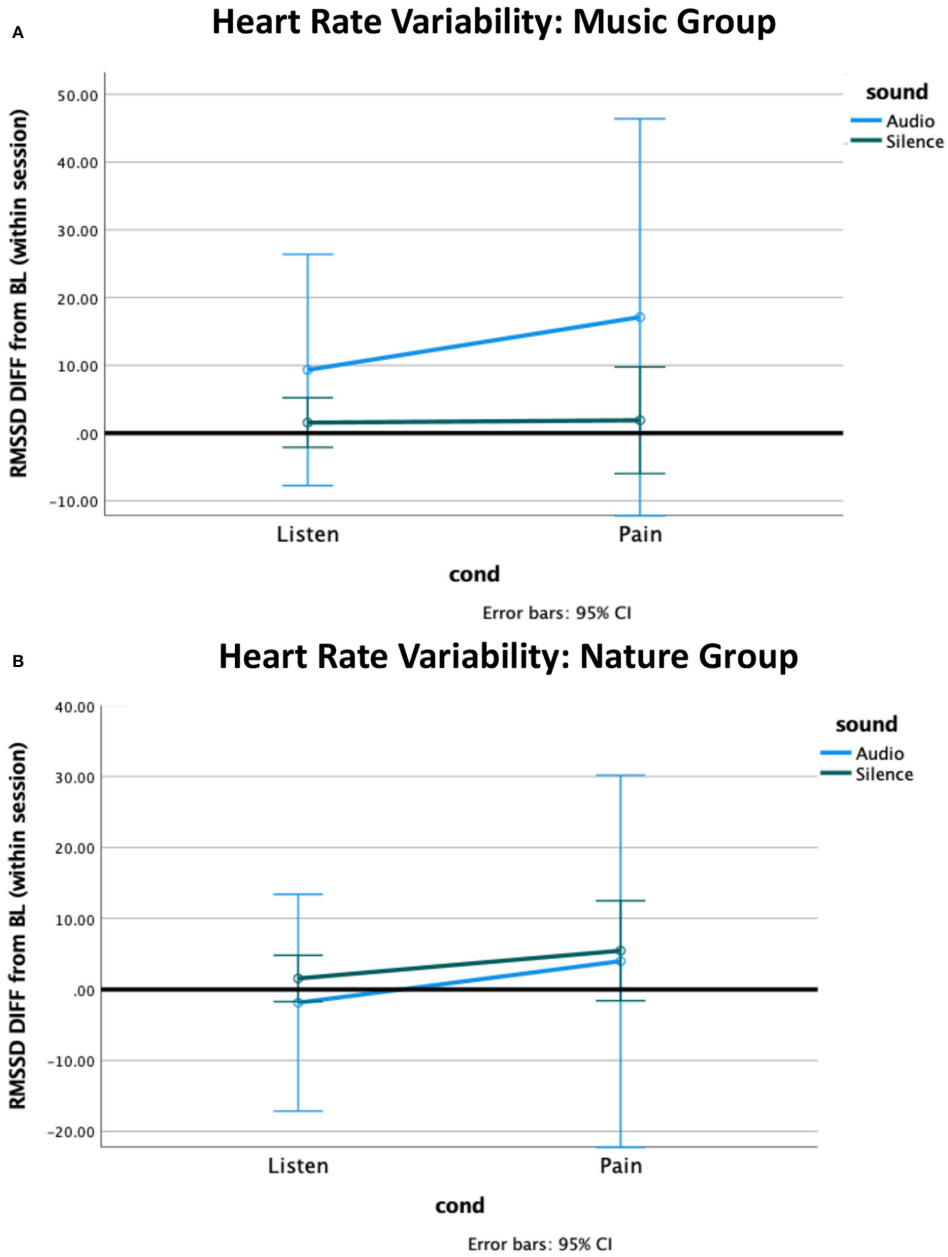
Heart rate variability (HRV) difference from within-session baseline. **(A)** The HRV in the Music group increased from baseline to the listening condition, and further increased during pain, with no effect observed for silence. **(B)** The Nature group had marginally greater HRV during pain. The HRV during pain was associated with a high standard deviation in both groups.

The independent samples Mann–Whitney *U*-test for heart rate revealed a non-significant trend for a group difference while Listening during Silence (*p* = 0.06), with the Nature group having slightly more reduced heart rate from baseline compared to the Music group (Figure 2). No other effects were significant. The non-parametric tests for heart rate variability (HRV) revealed no significant effects (Figure 3, Supplementary Table 3).

## Discussion

In this pilot study, we measured the analgesic effects associated with music and nature sounds on objective autonomic system responsiveness to painful stimuli. Our experimental design allowed for blinding during both data collection and analysis, reducing the potential for bias. By counterbalancing the order of audio presentation, we showed the feasibility of repeated measures testing in patients with FM while controlling for order effects. Even in our small sample, randomization successfully yielded relatively matched groups, with no group differences observed for FM symptoms, age, marital status, or education. By random chance, we did observe between-group differences in anxiety, although the Nature group was numerically only seven points higher than the Music group, and both groups were within one standard deviation of the standardized mean on the PROMIS scale.

### Objective pain

This study aimed to manipulate two potential mechanisms for music-evoked analgesia: cognitive distraction and physiological or vagal response alteration. By using musical stimuli and active control (Nature sounds), and comparing to each participant's own Silence control, our experimental design allows for the examination of distraction due to general relaxing audio, as well as examining music-specific analgesia by comparing the Music to the Nature Sound condition directly. We hypothesized that both listening conditions (Music and Nature) would reduce pain sensitivity compared to testing during silence and that music listening would reduce pain sensitivity compared to nature sounds (32, 34, 36).

#### Temporal summation

We observed a strong effect of cognitive distraction, with reduced temporal summation during either audio condition compared to silence, indicating that the auditory stimulus was effective in reducing pain. The direction of the group difference was opposite to our hypothesis, with the Nature group showing an analgesic effect, while the Music group showed none. This could be due to the confound of anxiety symptoms between the groups, or it could be a potential confound of pre-existing differences in sensitization between the groups, as temporal summation overall was somewhat higher in the Music group compared to the Nature group (20).

#### Mechanical pain tolerance

Interestingly, we did not observe any effects of group or condition on tolerance to thumb pressure. This was surprising, as this test usually shows high sensitivity for variations in pain response (22). However, it is possible that the transient changes between sessions were too small to be observed in this small sample, and that a larger sample or longer intervention would be necessary to see differences in maximal pain tolerance.

### ANS

#### Heart rate

We also hypothesized that music would increase vagal input to the autonomic nervous system, decreasing heart rate and increasing heart rate variability compared to both silence and nature sounds. Vagal response during pain is a coping mechanism (65). We did observe a small difference between the groups in heart rate pointing to the feasibility of the chosen stimuli, yet the direction was opposite to our hypothesis with the Nature group having greater reductions from baseline compared to the Music group. This could also be related to group differences in anxiety or other pre-existing physiological differences between the groups. The Nature group, having higher anxiety (*p* = 0.02), could have had elevated heart rate at baseline, thereby having more chance for the analgesic effect to be observed. In our analysis, we corrected for the within-session baseline to address this possibility.

#### Heart rate variability

Heart rate variability is a better measure for vagal response than raw heart rate (66). However, we observed no significant effects for HRV, suggesting that we were underpowered to observe a vagal response with this small sample. While not significant, the Music group did show a pattern of response that was consistent with vagal activation similar to other studies, with a reduction from baseline and then further reduction during pain, that was not observed in the Nature group (67, 68). Such anticipated response might be due to emotional expression toward the music stimulus, enjoyment, or simply just being entertained, an effect that might have been increased had the participants selected the music themselves. A larger sample would be needed to clarify whether there is a greater vagal response to music more generally.

### Individual differences

Individual differences likely play a role in how a person will respond to auditory stimulation (69–71). While we measured many of these potential differences, including fibromyalgia symptoms, mood symptoms, and music experience, our small sample size did not allow for comparisons between them. However, these are likely important variables to consider in future trials.

## Limitations

This study is limited by the small sample size, and the results should be interpreted with caution. Due to the small sample size, it was difficult to fully balance the groups. Our groups differed on anxiety potentially confounding our results, although all participants were in the mild to moderate anxiety range. Additionally, although it was not statistically significant, more participants in the Music group had attained education beyond a bachelor's degree. However, inherently when having a small sample size, it is somewhat easier to detect within-participant effects rather than between-participant effects. It is possible that a greater analgesic effect would be elicited from the music of an individual's choice, as that could potentially have greater associations with positive memories and previous experience, thus enhancing the physiological response. Our experimental design using nature sounds as an auditory control and carefully selected musical selections with characteristics hypothesized to facilitate relaxation and analgesia is a strength, and can be used in future studies to separate the effects of auditory distraction from music-specific effects.

## Conclusion

In conclusion, our current results did not support our hypothesis of stronger analgesic effects of music vs. distracting nature sounds; however, we did observe strong effects of auditory distraction on pain temporal summation and tolerance. The confounding effect of anxiety symptoms in our study, as well as the individual differences observed on the MEQ, suggest that variability in mood and other factors may be important in understanding how individuals will respond to music or other auditory stimuli to gain therapeutic analgesic effects. While these results should be treated with caution, this study provides preliminary evidence that some individuals may benefit from music or audio stimulation as a treatment more than others. Further study is warranted.

## Data availability statement

The datasets presented in this study can be found in online repositories. The names of the repository/repositories and accession number(s) can be found at: https://osf.io/84baz.

## Ethics statement

The studies involving human participants were reviewed and approved by University of Kansas Medical Center Institutional Review Board Human Subjects Committee. The patients/participants provided their written informed consent to participate in this study.

## Author contributions

RL: conceptualization, methodology, formal analysis, investigation, data curation, writing of the original draft, project administration, and funding acquisition. MM: formal analysis, investigation, data curation, reviewing and editing, and project administration. AC: conceptualization, methodology, resources, writing of the original draft, reviewing and editing, and funding acquisition. ZM: reviewing and editing. LM: resources and reviewing and editing. KG: conceptualization, methodology, investigation, resources, and reviewing and editing. All authors contributed to the article and approved the submitted version.

## Funding

This study was funded by the National Institutes of Health through a KUMC Frontiers Arts+Medicine Trailblazer to RL (NIH UL1TR002366), NIH K23GM123320 to AC, and by the KUMC Hoglund Biomedical Imaging Center (HBIC Neurophysiology Core and Cognitive Neuroscience Unit). The Hoglund Biomedical Imaging Center is supported by a generous gift from Forrest and Sally Hoglund and funding from the National Institutes of Health including: UL1 TR002366: Frontiers: KU Institute for Clinical & Translational Science, and a KUMC Equipment Grant.

## Conflict of interest

AC serves as a consultant for Swing Therapeutics. The remaining authors declare that the research was conducted in the absence of any commercial or financial relationships that could be construed as a potential conflict of interest.

## Publisher's note

All claims expressed in this article are solely those of the authors and do not necessarily represent those of their affiliated organizations, or those of the publisher, the editors and the reviewers. Any product that may be evaluated in this article, or claim that may be made by its manufacturer, is not guaranteed or endorsed by the publisher.
