# Supplementary material for: Autonomic nervous system markers of music-elicited analgesia in people with fibromyalgia: A double-blind randomized pilot study
Source: Front Pain Res (Lausanne). 2022 Sep 15;3:953118. doi: 10.3389/fpain.2022.953118 (PMC9521378; doi:10.3389/fpain.2022.953118)

Supplementary Material


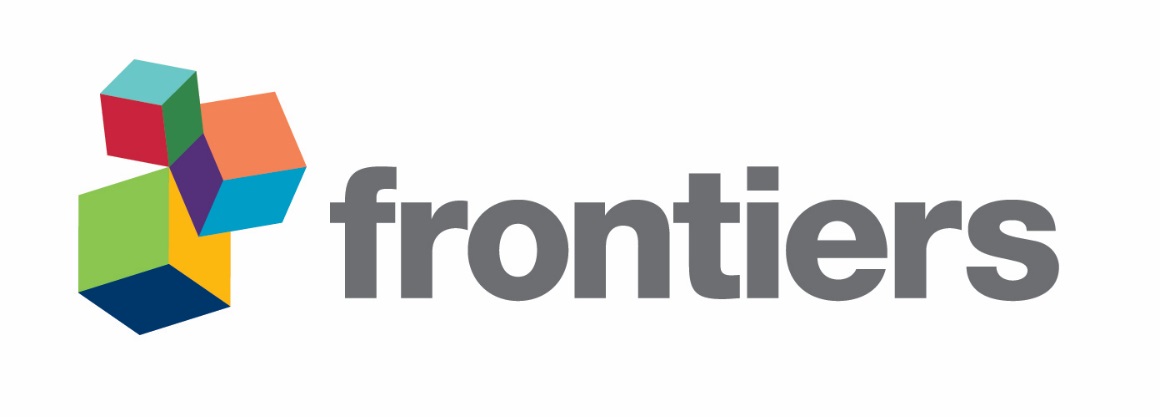


**Supplementary Table 1** Audio recordings used during the listening conditions.

| Year | Title | Composer | Recording Artist | Album |
| --- | --- | --- | --- | --- |
| Nature Sounds | | | | |
| 2011 | Gentle Birds and Forest Stream for Relaxation Meditation | N/A | N/A | Bird Sounds – Morning Birds for Relaxation, Meditation, Yoga, Naturescapes, Forest Ambience and Spa |
| 2011 | A Forest Ambience – Nocturne Nature Sounds Mother Earth Sleep Music for Relaxation, Meditation, Massage, Toga Class, Tai Chi, Reiki, Tantra and Zen | N/A | N/A | The Secret Garden Nature Sounds – Relaxing Sounds of Nature for Deep Sleep, Baby Sleep, Yoga Pregnancy, Yoga Music Relaxation |
| Western Classical Music | | | | |
| 2017 | Deux arabesques, L. 66: No. 1 in E Major, Andantino con moto | Debussy, Claude | Battel, Giovanni Umberto | Classical Music Collection |
| 2019 | Bagatelle No. 25 in A Minor, WoO 59 “Für Elise” | Beethoven, Ludwig van | Battel, Giovanni Umberto | 50 Most Famous Pieces of Classical Music |
| 2019 | The Four Seasons, Violin Concerto No. 4 in F Minor, RV 297 “Winter”: II. Largo | Vivaldi, Antonio | Pavel Lyubomudrov, Metamorphose String Orchestra, Yuliya Lebedenko Conductor | Baroque Music for Studying & Brain Power |
| 2017 | Piano Sonata No. 14 in C-Sharp Minor, Op. 27, No. 2 “Moonlight Sonata”: I. Adagio sostenuto | Beethoven, Ludwig van | Battel, Giovanni Umberto | Sad, Melancholic Classical Music |
| 2017 | Symphony No. 4 in A Major, Op. 90 “Italian”: II. Andante con moto | Mendelssohn, Felix | Orchestra da Camera Florentina, Giuseppe Lanzetta Conductor | Romantic Music – Classical Music from the Romantic Period |

**Supplementary Table 2** Primary outcome variables tests of non-normal distribution.

| Measure | Mean (SD) | Skewness (SE) | Kurtosis (SE) |
| --- | --- | --- | --- |
| Music Group (n=4) | | | |
| Temporal Summation - Audio | 20.25 (14.29) | 0.10 (1.01) | -5.39 (2.62) |
| Temporal Summation - Silence | 20.17 (13.14) | 0.04 (1.01) | -5.74 (2.62) |
| Mechanical Pain Tolerance - Audio | 4.12 (1.02) | 0.57 (1.01) | -2.00 (2.62) |
| Mechanical Pain Tolerance - Silence | 4.18 (1.00) | -1.58 (1.01) | 2.54 (2.62) |
| HR: Listen, Audio | -2.81 (1.78) | -1.25 (1.01) | 1.95 (2.62) |
| HR: Listen, Silence | -2.37 (0.69) | -1.87 (1.01) | 3.53 (2.62) |
| HR: Pain, Audio | -4.44 (1.30) | 0.17 (1.01) | -4.58 (2.62) |
| HR: Pain, Silence | -3.43 (1.92) | -1.21 (1.01) | 0.91 (2.62) |
| HRV: Listen, Audio | 9.33 (21.46) | 1.36 (1.01) | 2.02 (2.62) |
| HRV: Listen, Silence | 1.55 (3.71) | -0.36 (1.01) | 0.72 (2.62) |
| HRV: Pain, Audio | 17.11 (37.67) | 1.93 (1.01) | 3.74 (2.62) |
| HRV: Pain, Silence | 1.89 (6.73) | -0.42 (1.01) | -2.27 (2.62) |
| Nature Group (n=5) | | | |
| Temporal Summation - Audio | 4.13 (5.60) | 1.29 (0.91) | 0.70 (2.00) |
| Temporal Summation - Silence | 9.40 (7.44) | 1.92 (0.91) | 4.04 (2.00) |
| Mechanical Pain Tolerance - Audio | 5.17 (0.85) | 0.16 (0.91) | -2.32 (2.00) |
| Mechanical Pain Tolerance - Silence | 5.05 (0.94) | 0.85 (0.91) | 0.30 (2.00) |
| HR: Listen, Audio | -3.37 (2.85) | 0.28 (0.91) | -2.29 (2.00) |
| HR: Listen, Silence | -4.46 (1.33) | 0.59 (0.91) | -2.66 (2.00) |
| HR: Pain, Audio | -4.33 (0.95) | -0.14 (0.91) | -1.63 (2.00) |
| HR: Pain, Silence | -6.77 (5.60) | -1.29 (0.91) | 1.48 (2.00) |
| HRV: Listen, Audio | -1.87 (4.41) | 0.36 (0.91) | -0.38 (2.00) |
| HRV: Listen, Silence | 1.56 (2.52) | -1.03 (0.91) | 1.79 (2.00) |
| HRV: Pain, Audio | 3.99 (3.19) | 0.29 (0.91) | -1.00 (2.00) |
| HRV: Pain, Silence | 5.46 (6.60) | 1.06 (0.91) | 1.32 (2.00) |

**Supplementary Table 3** Heart rate variables corrected for within session baseline by condition (Listen, Pain), session (Audio, Silence), and audio group assignment (Music, Nature).

|  | Music Group (*n* = 4)  (*M* (*SD*)) | Nature Group (*n* = 5)  (*M* (*SD*)) | Statistical Test | *Z (p)* |
| --- | --- | --- | --- | --- |
| HR_BL_: Cond. = Listen  Session = Audio | -2.81 (1.78) | -3.37 (2.85) | -- | -- |
| HR_BL_: Cond. = Pain  Session = Audio | -4.44 (1.30) | -4.33 (0.95) | -- | -- |
| HR_BL_: Cond. = Listen  Session = Silence | -2.37 (0.69) | -4.46 (1.33) | -- | -- |
| HR_BL_: Cond. = Pain  Session = Silence | -3.43 (1.92) | -6.72 (5.60) | -- | -- |
| Group Difference:  Listen: Silence | -- | -- | Independent Samples Mann-Whitney U | 2.00 (.06^†^) |
| Group Difference:  Listen: Audio | -- | -- | Independent Samples Mann-Whitney U | 10.00 (1.00) |
| Group Difference:  Pain: Silence | -- | -- | Independent Samples Mann-Whitney U | 6.00 (.41) |
| Group Difference:  Pain: Audio | -- | -- | Independent Samples Mann-Whitney U | 10.00 (1.00) |
| Session Difference:  Pain minus Listen: Audio versus Silence | -- | -- | Related-Samples Wilcoxon Signed-Rank | 21.00 (.86) |
| Group Difference in  Pain minus Listen: Silence | -- | -- | Independent Samples Mann-Whitney U | 9.00 (.91) |
| Group Difference in  Pain minus Listen: Audio | -- | -- | Independent Samples Mann-Whitney U | 12.00 (.73) |
| Group Difference in Pain minus Listen: Between Session Change | -- | -- | Independent Samples Mann-Whitney U | 13.00 (.41) |
| HRV_BL_: Cond. = Listen  Session = Audio | 9.33 (21.46) | -1.87 (4.41) | -- | -- |
| HRV_BL_: Cond. = Pain  Session = Audio | 17.11 (37.67) | 3.99 (3.19) | -- | -- |
| HRV_BL_: Cond. = Listen  Session = Silence | 1.55 (3.71) | 1.56 (2.52) | -- | -- |
| HRV_BL_: Cond. = Pain  Session = Silence | 1.89 (6.73) | 5.46 (6.60) | -- | -- |
| Group Difference:  Listen: Silence | -- | -- | Independent Samples Mann-Whitney U | 11.00 (1.00) |
| Group Difference:  Listen: Audio | -- | -- | Independent Samples Mann-Whitney U | 7.00 (.56) |
| Group Difference:  Pain: Silence | -- | -- | Independent Samples Mann-Whitney U | 12.00 (.73) |
| Group Difference:  Pain: Audio | -- | -- | Independent Samples Mann-Whitney U | 12.00 (.73) |
| Session Difference:  Pain minus Listen: Audio versus Silence | -- | -- | Related-Samples Wilcoxon Signed-Rank | 13.00 (.26) |
| Group Difference in  Pain minus Listen: Silence | -- | -- | Independent Samples Mann-Whitney U | 12.00 (.73) |
| Group Difference in  Pain minus Listen: Audio | -- | -- | Independent Samples Mann-Whitney U | 13.00 (.56) |
| Group Difference in Pain minus Listen: Between Session Change | -- | -- | Independent Samples Mann-Whitney U | 15.00 (.29) |

**Notes:** Between Session heart rate and heart rate variability measures were assessed with Related-Samples Wilcoxon Signed-Rank Tests. Between Group comparisons were assessed with Independent Samples Mann-Whitney U Tests. Within Session Change Scores were calculated as Pain minus Listen, and between Session Change Scores were calculated per participant as Audio minus Silence. Both were compared Between Groups with Independent Samples Mann-Whitney U Tests. ^†^Indicates non-significant effects at *p*<.10.

**Abbreviations:** M, mean; SD, standard deviation; HR_BL_, heart rate baseline corrected; Cond., condition; HRV_BL_, heart rate variability baseline corrected: root mean square of successive differences between heartbeats baseline corrected

**Supplementary Figure 1** Histograms for distribution of primary outcome variables during Silence and Audio sessions of A) Temporal Summation, B) Mechanical Pain Tolerance, C) Heart Rate during Listening and Pain, and D) Heart Rate Variability during Listening and Pain. The distribution for most variables is non-normal.
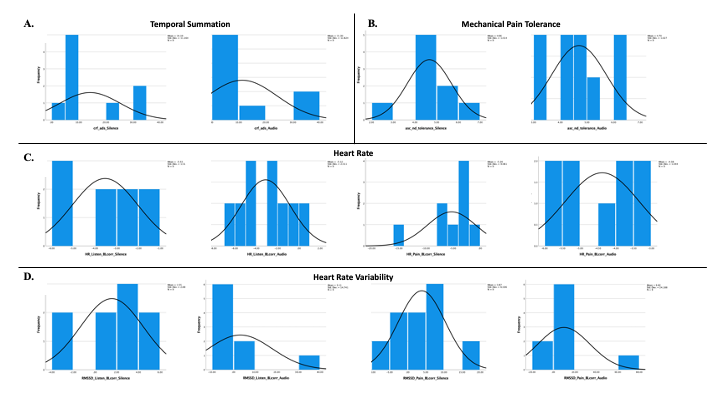

Supplement: Supplementary file 1 [file Data_Sheet_1.docx]
